# Gut-reproduction axis: a new perspective on understanding pelvic inflammatory disease

**DOI:** 10.3389/fmicb.2026.1822640

**Published:** 2026-08-10

**Authors:** Shuai Ma, Min Wang, Xiao Tan, Caomeihui Shen, Xiaorui Tao, Minjia Sheng, Xueying Zhang, Ying Xu, Lianwen Zheng

**Affiliations:** 1 Department of Obstetrics and Gynecology, The Second Hospital of Jilin University, Changchun, China; 2 The Reproductive Medical Center, China-Japan Union Hospital of Jilin University, Changchun, China; 3 Clinical Medicine Program, Xinjiang Medical University, Ürümqi, China; 4 College of Animal Science, Jilin University, Changchun, China; 5 Jilin Guojian Economic Development Obstetrics and Gynecology Hospital, Changchun, China

**Keywords:** etiology, gut microbiota, human microbiome, infertility, metabolomics, pelvic inflammatory disease, therapeutics

## Abstract

When exploring the macro trend of declining global fertility rates, a pathological factor often hidden beneath individual suffering cannot be ignored: pelvic inflammatory disease (PID). Recurrent inflammation leads to tissue damage, adhesions, and scaring in the fallopian tubes, ovaries, and even the entire pelvic cavity, ultimately causing serious pelvic organ sequelae that severely affect female quality of life. The clinical diagnosis and treatment of PID are very challenging, with issues such as inappropriate treatment plan selection, insufficient treatment duration, and poor patient compliance. The role of microecological balance in maintaining human health has been increasingly recognized. Human tissues and organs form a dynamic, interactive system with microorganisms, playing an important role in female reproductive function. As an important microecological system of the body, the reproductive tract has a relatively complete natural defense function to resist infection. Previous research on the microbiome and female reproductive health has rarely focused on PID. The gut–reproduction axis aims to construct an integrated regulatory model in which the homeostasis of the gut microbiota plays a crucial role in reproductive health. The microbiota affects the occurrence of PID by regulating core pathways, such as hormone secretion, inflammatory responses, and immune homeostasis. Examining the effects of microecologies in animal models and preliminary clinical studies will lay a solid theoretical foundation for developing microbiota-targeted innovative diagnostics and treatment strategies for PID, improving female reproductive health throughout their life course.

## 1 Introduction

Pelvic inflammatory disease (PID) is a common infectious disease of the female upper reproductive tract (Brunham et al., 2015; Wiesenfeld et al., 2021). Due to the concealment of early symptoms or incomplete treatment, PID has become an important cause of tubal adhesions and blockages, directly depriving countless women of their natural fertility. PID may cause ovarian cancer and adversely affect patients’ fertility and quality of life, seriously impacting female reproductive health and increasing the family and socioeconomic burden on patients (Greydanus et al., 2018). Studies have reported that the female reproductive tract (FRT) represents a microbial continuum, with the relative abundance of *Lactobacillus* spp. gradually decreasing from the lower to the upper reproductive tract (Chen et al., 2017). When the defense mechanisms of the reproductive tract are compromised or immune function declines, the natural protective barrier is weakened, allowing pathogenic bacteria to ascend from the lower to the upper reproductive tract, ultimately leading to PID (Causer et al., 2022). The human microbiome comprises all microorganisms and their genetic material residing in the body, playing a key role in regulating immunity, maintaining metabolic balance, and other functions (Porcari et al., 2025). The FRT, as an important micro-ecosystem in the human body, exhibits interactions and causal relationships between dysbacteriosis of the reproductive tract and gynecological diseases (Koedooder et al., 2019). The gut–reproduction axis may contribute to the pathogenesis of PID through multiple pathways, including immune modulation, inflammatory response, and endocrine interactions.

## 2 Overview of PID

**FIGURE 1:**
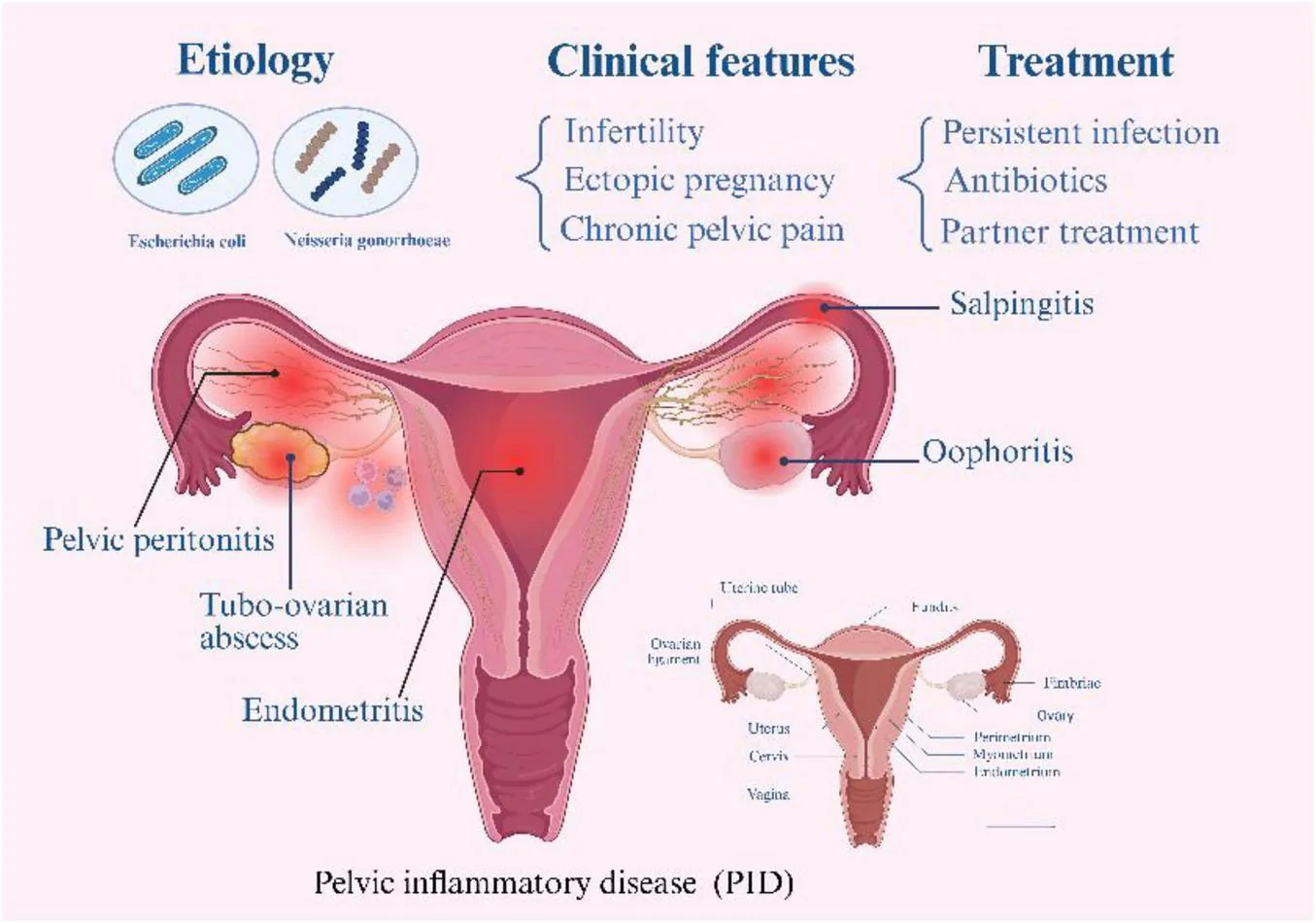
PID. PID primarily includes endometritis, salpingitis, tubo-ovarian abscesses, and pelvic peritonitis, with salpingitis being the most common. This pathological condition can lead to serious clinical complications, including infertility, ectopic pregnancy, and chronic pelvic pain. The standard clinical management of PID relies primarily on antibiotic therapy and concurrent treatment of sexual partners. Created with BioRender.

PID refers to a group of diseases caused by infections of the upper FRT, mainly including endometritis, salpingitis, salpingo-oophoritis, tubo-ovarian abscess, and pelvic peritonitis (Bartlett et al., 2013). In recent years, the incidence of PID has shown an increasing trend among adolescents, becoming a significant public health issue worldwide (Greydanus and Dodich, 2015; Greydanus et al., 2022; Figure 1). Infections by pathogens such as bacteria, *Chlamydia* spp., and *Mycoplasma* spp. are often related to the occurrence of PID (Yagur et al., 2021). Taylor et al. found that the main pathogens of PID in the United States are *Neisseria gonorrhoeae*, *Chlamydia* spp., and *Mycoplasma* spp. (Taylor et al., 2011). Xholli et al. found that the main pathogen of PID in Italy is *Chlamydia* spp., while the detection rates of *Mycoplasma* spp. and *Neisseria gonorrhoeae* are low (Xholli et al., 2014). The FRT microbiota is a dynamically balanced system, that plays an important role in preventing reproductive tract infections by forming a membrane barrier (Ravel et al., 2011). Bacterial co-infections in the FRT alter the microecological balance, disrupt the stability of the microbiota, and lead to the occurrence of PID.

### 2.1 Pathophysiology of PID

The occurrence and progression of PID are related to inflammatory responses and immune function (Mitchell and Prabhu, 2013). The development of PID includes three stages: pathogenic microorganism infection, activation of the host immune system, and the release of anti-infective factors. The inflammatory response is an important link in the progression of PID (Gao et al., 2025). IL-1β, as a pro-inflammatory factor, is positively correlated with PID, and IL-1β alters the pelvic environment, exacerbating inflammatory and tissue damage in the pelvic (Lee et al., 2008). Both IL-2 and IL-6 are inflammatory factors produced by the body in response to inflammatory damage. IL-6, as a key factor initiating the inflammatory response, is mainly produced by monocytes and macrophages. Research has found that serum C-reactive protein may support the diagnosis of PID, although it is non-specific and can be normal in mild or moderate cases (Ross et al., 2014). Tumor necrosis factor-α (TNF-α) causes inflammatory cells to aggregate at the lesion site in PID, exacerbating the inflammatory response (Xu et al., 2025).

As a group of infectious diseases, PID shows a positive correlation with immune dysfunction (Darville, 2021). Women over the age of 40 have a higher risk of recurrent PID compared to young women, which may be related to immune dysfunction (Safrai et al., 2020). Toll-like receptors (TLRs) recognize microbial components after they breach physical barriers and play an important role in host defense against genital tract infections (Sonnex, 2010). Dysregulated TLR expression may contribute to either insufficient pathogen clearance or excessive inflammatory responses, potentially leading to tissue damage. TLRs are a key factor linking bacterial infection to the occurrence of inflammation, and their function is mainly achieved through the TLRs NF-κ B signaling pathway, which is the core molecular mechanism regulating the expression and release of inflammatory mediators (Taylor et al., 2013).

### 2.2 The clinical presentation and classification of PID

The clinical manifestations of PID vary widely, some patients are asymptomatic, while others experience clinical features such as lower abdominal pain, abnormal uterine bleeding, difficulty urinating, and fever (Soper, 2010). Long-term inflammation in pelvic tissues leads to tissue fibrosis and adhesion, which in turn causes structural and functional abnormalities of the fallopian tubes, resulting in irreversible reproductive damage (Murray et al., 2021). Recurrent and persistent PID leads to inflammatory complications such as diffuse peritonitis and sepsis (Nkwabong and Dingom, 2015). PID patients often experience negative emotions such as depression and anxiety, which seriously affect their quality of life and physical and mental health (Shen et al., 2016). Research has shown a potential positive correlation between depression states in adult women and the prevalence of PID (Huang et al., 2022). Gynecological examination reveals abnormal vaginal discharge, which is yellow and mucous-like, with some accompanied by a foul odor. There may be obvious congestion of the cervix or vaginal walls. In severe cases, signs of peritoneal irritation are positive. During gynecological examination, most patients with tenderness in the uterus or adnexal area and cervical motion tenderness.

Clinically, PID is divided into acute and chronic types (Ha et al., 2019). Acute PID is caused by pathogenic microorganisms entering the endometrium and tissues adjacent to the fallopian tubes through the vagina, leading to local uterine microcirculation disorders, compression of nerve fibers, and symptoms such as abdominal pain. Chronic PID often results from acute PID that has not been thoroughly treated, leading to a prolonged course and recurrent attacks (Siegenthaler et al., 2020). There are also some specific types of PID, such as Fitz-Hugh-Curtis syndrome (You et al., 2012), pregnancy/lactation PID (Mackeen et al., 2015), PID with an intrauterine device (Tepper et al., 2013), female genital tuberculosis PID (Yates et al., 2017), and pelvic actinomycosis PID (Nakahira et al., 2017).

### 2.3 The therapeutic bottleneck in PID

The clinical presentation of PID is variable. Many affected women have mild or no apparent symptoms, and a wide range of pathogens may be involved. Coupled with the special anatomical structure of the pelvis, the clinical diagnosis and treatment of PID are challenging (Savaris et al., 2020; Taira et al., 2022). Untreated PID may increase the transmission of human immunodeficiency virus (Kreisel et al., 2021). If cervical secretions appear normal and no white blood cells are observed in vaginal secretion microscopy, indicating a low likelihood of PID, with a negative predictive value of 95% and a positive predictive value of only 17% (Yudin et al., 2003). Laparoscopy provides a more accurate and comprehensive diagnosis, but its accuracy is lower for mild or asymptomatic PID (Jenkins and Vadakekut, 2025). PID needs to be differentiated from ectopic pregnancy, ovarian cyst torsion, acute appendicitis, endometriosis, and inflammatory bowel disease (Ross et al., 2018). Due to the unclear etiology and microbial characteristics of PID, the treatment measures for patients are not standardized. Currently, clinical treatment of PID mainly focuses on restoring the balance of the microbial environment at the lesion site, usually through medication. Antibiotic treatment has achieved certain effects in treating PID (Savaris et al., 2019). A 2016 gonococcal antimicrobial surveillance program reported that the high resistance of *Neisseria gonorrhoeae* to azithromycin is becoming increasingly severe worldwide (Day et al., 2018).

The pathogens that cause PID are complex, and there may be overlapping mixed infections. Although potent anti-infective treatment quickly alleviates symptoms, long-term treatment with broad-spectrum and high-dose antibiotics not only easily leads to bacterial resistance and secondary infections but also increases the risk of adverse reactions (Den Heijer et al., 2020). The balance of pelvic microbiota is disrupted, the defense function is reduced, and the body’s barrier against pathogenic bacteria weakens, further exacerbating microecological imbalance. Microbial imbalance affects the interactions within the host -microbiota-immune-inflammation axis, influencing host physiological or pathological processes.

Clarifying the etiology and microbial characteristics of PID, preventing its occurrence, and providing thorough treatment are key and challenging aspects of clinical research. The regulatory mechanisms of the gut–reproduction axis integrate microbial signals into the pathophysiological network of PID by constructing a novel theoretical framework, providing a new perspective for elucidating PID etiology and laying a theoretical foundation for developing innovative diagnostic and therapeutic strategies based on the microbiome.

## 3 Microbiome

The human microbiome is a complex and diverse community of microorganisms, composed of both symbiotic and pathogenic microbes (de Vos et al., 2022; Martino et al., 2022). These microbial communities typically colonize the gastrointestinal tract, pelvic cavity, abdominal cavity, reproductive tract, and brain, including bacteria, fungi, eukaryotes, and viruses. The sum of genetic information of all these microorganisms is referred to as the microbiome (Lloyd-Price et al., 2016). The microbiome is considered a functional entity with endocrine and immune regulatory properties, rather than merely a collection of bacteria (Geva-Zatorsky et al., 2017). The human microecological system generates a series of small-molecule metabolites, such as short-chain fatty acids (SCFAs) and secondary bile acids, through complex metabolic networks. These metabolites act as key signaling molecules and enter the circulatory system, regulating the host through pathways such as G protein-coupled receptors, affecting immune balance and endocrine homeostasis, and widely participating in various physiological and pathological processes (Chadchan et al., 2021). Microbial metabolites not only maintain the host’s immune balance but also intervene in endocrine functions, regulating hormone synthesis, metabolism, and signal transduction, and widely participate in the occurrence and development of various systemic diseases in the body (Garrett, 2015; Sen et al., 2021). The interaction between microorganisms and hosts involves the exchange of energy, substances, and genetic information, forming a dynamic balance that is both coordinated and constrained by each other. The occurrence of PID may be associated with microbial imbalance. When the body is affected by endogenous or exogenous factors, disruption of the microbial balance may contribute to disease pathogenesis, although it is likely one of multiple contributing factors, including host, microbial, behavioral, and environmental influences (Barrientos-Durán et al., 2020).

### 3.1 Gut microbiota

**FIGURE 2:**
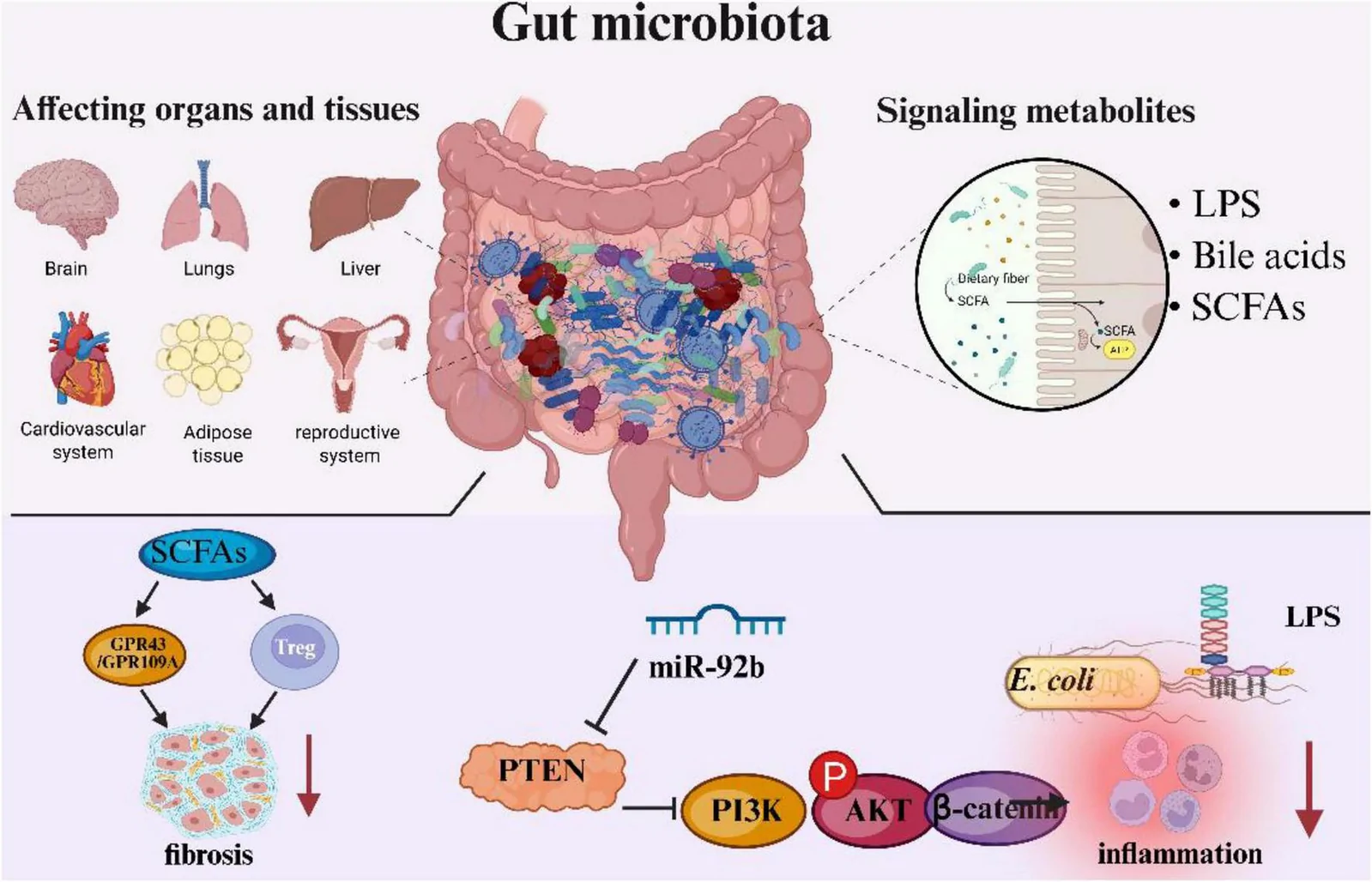
Gut microbiota and diseases. GM produces a series of metabolites, including LPS, bile acids, and SCFAs. SCFAs exert protective biological functions by activating GPR43/GPR109A receptors and promoting Treg cell-mediated immune tolerance, thereby alleviating tissue fibrosis lesions. LPS increases miR-92b expression to inhibit PTEN activity, followed by inducing phosphorylation-dependent activation of the PI3K/AKT/β-catenin signaling cascade. Created with BioRender.

The gut microbiota (GM) is a complex microbial ecosystem that exists in both humans and animals (Chen et al., 2021). There are more than 1000 species of bacteria in the human GM, totaling over 100 trillion (Saad et al., 2016). Due to their vast numbers and powerful functions, GM is known as the human “second genome” (Zhao et al., 2023). Under the combined influence of the host and external factors, GM is in a stage of dynamic equilibrium, and its effects on the host change with the host’s environment. In GM, nearly 90% are Firmicutes and Bacteroidetes, followed by Actinobacteria and Proteobacteria (Qin et al., 2010). GM is involved in multiple physiological processes, including the synthesis of SCFAs and vitamins, as well as the metabolism of lipids and bile acids (Yang et al., 2021). GM and its metabolites are key to building the chemical barrier. The metabolites produced by GM are crucial for immune regulation and anti-inflammatory function, maintaining intestinal homeostasis, and effectively preventing harmful substances, such as bacteria and endotoxins, from entering the blood circulation and other tissues (Zhang et al., 2020). Disruption of the GM may impair the mucosal barrier, leading to increased systemic lipopolysaccharide (LPS) and activation of inflammatory pathways, which can affect immune and metabolic homeostasis (Aljadah and Widlansky, 2023). Whether these changes contribute to reproductive system dysfunction requires further study. MiR-92b is activated by monocytes when stimulated by specific pathogen-associated molecular marker LPS from *Escherichia coli*, inhibiting the TLR4 signaling pathway via the PI3K/AKT/β-catenin axis and suppressing the production of pro-inflammatory factors TNF-α and IL-6 (Jiang et al., 2021; Figure 2).

### 3.2 Microbiome of the FRT

**FIGURE 3:**
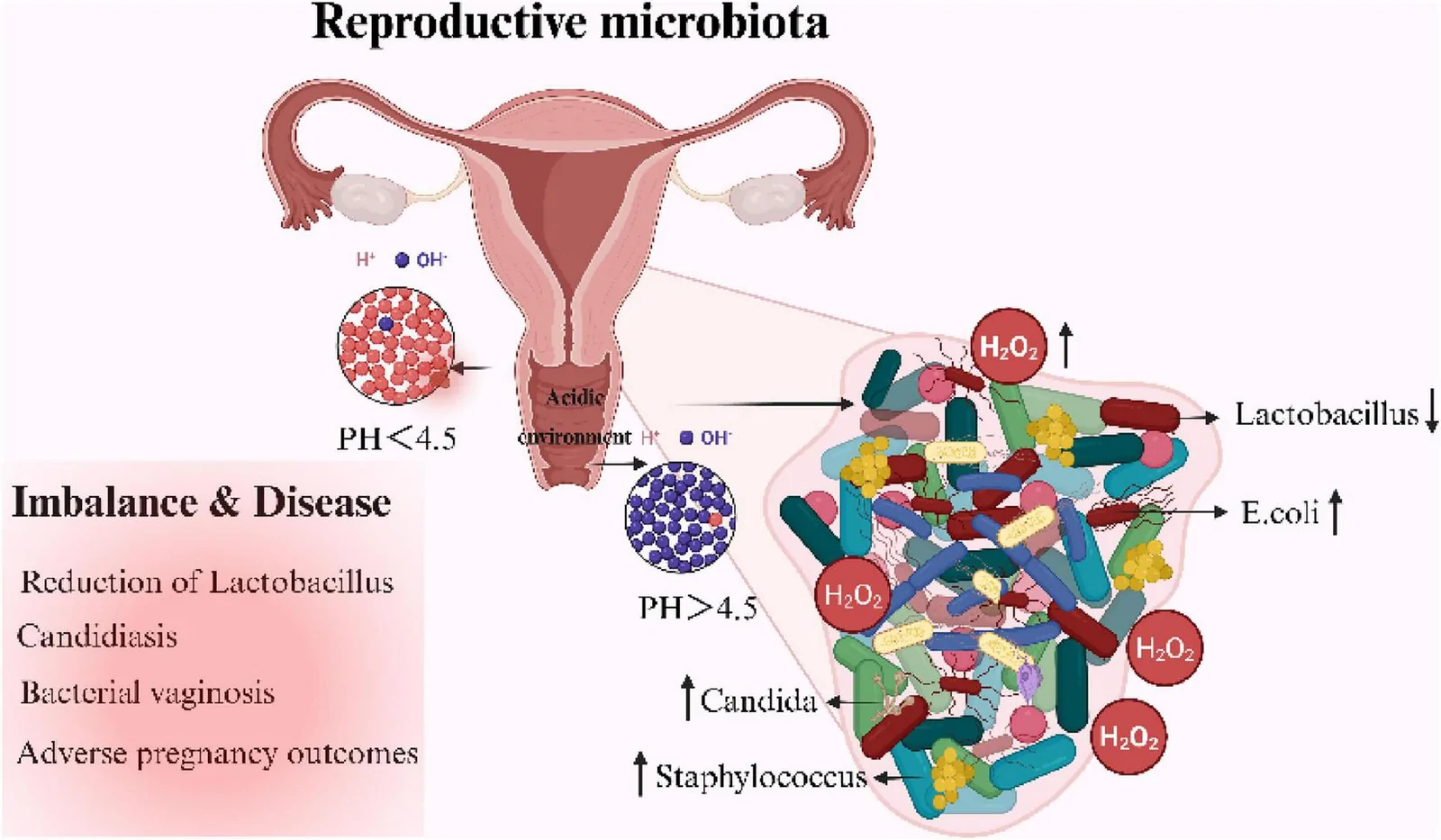
Imbalance of reproductive microbiota and diseases. The healthy FRT is predominantly colonized by *Lactobacillus*, which secrete hydrogen peroxide and hydrogen ions to sustain an acidic luminal microenvironment with pH below 4.5 and establish defensive mucosal homeostasis against pathogenic invasion. Disruption of reproductive microbial homeostasis triggers the depletion of protective *Lactobacillus*, accompanied by overproliferation of opportunistic pathogens including *E. coli*, *Candida*, and *Staphylococcus*, which elevates vaginal pH above 4.5 and eliminates the microbicidal acidic barrier. Such reproductive dysbiosis sequentially increases the susceptibility to multiple clinical complications, including candidiasis, bacterial vaginosis, and various adverse pregnancy-related outcomes. Created with BioRender.

The FRT microecology is an important component of the human microecology, and changes in reproductive tract microecology are one of the important reasons affecting reproductive health (Torcia, 2019; Figure 3). The FRT is divided into the upper and lower reproductive tracts, with the upper reproductive tract including the cervical canal, uterus, fallopian tubes, and ovaries, and the lower reproductive tract including the vagina and cervical dilation. A continuous microbial community colonizes the FRT, and it has been reported that the female upper reproductive tract is not a “sterile” environment (Chen et al., 2017; Wee et al., 2018). The number of bacteria in the upper reproductive tract is much lower than that in the vagina, but the bacterial diversity is higher than that in the vagina (Fang et al., 2016). The microbiota of the reproductive tract forms a dynamic balance of mutual restraint and coordination with the host and environment, playing an important role in maintaining female reproductive health (Deka et al., 2021). The microbiome of the FRT is influenced by various factors, including race, environment, menstrual cycle, sexual activity, medications, etc. (Loeper et al., 2018; Noyes et al., 2018). Hormone levels are the most important influencing factor, and the microbial communities changed significantly in puberty, menstrual period, pregnancy, and menopause (Kim et al., 2021a). Research has found that upregulation of estrogen expression, external interventions, excessive sexual activity, and vaginal delivery in women of childbearing age all affect vaginal pH and increase the risk of vaginal microbiota imbalance.

The composition of the FRT microbiota varies, and acidifying the vaginal environment by producing organic acid metabolites is a common protective function (Gajer et al., 2012). In healthy women, the microbiota of the vagina, cervix, and uterine cavity is dominated by *Lactobacillus* spp., which are the core factors in maintaining the ecological balance of the microbiota (White et al., 2011). *Lactobacillus* spp. uses lactic acid, hydrogen peroxide, and bacterial toxins to resist or eliminate harmful microorganisms and inhibit the growth of pathogens (van de Wijgert et al., 2014). *Lactobacillus* spp. has a strong affinity with host cells, preventing the adhesion and colonization of other pathogenic bacteria. *Lactobacillus* spp. effectively inhibit the infectivity of *Chlamydia trachomatis*, reduce the survival rate of *Neisseria gonorrhoeae*, and inhibit the activity of *Escherichia coli* (Tamarelle et al., 2019). When the levels of *Lactobacillus* spp. decrease and loses its dominant position, other pathogens proliferate rapidly, leading to microbial imbalance and resulting in vaginitis. Bacterial ascending infection causes upper reproductive tract inflammation and damage to pelvic tissues (Lykke et al., 2021). When the balance of the reproductive tract microbiota is disrupted, the mucosal barrier function is impaired, increasing the risk of invasion by other microorganisms, which adds to the suffering of PID patients and prolongs the treatment cycle (Liang et al., 2023).

### 3.3 Gut-reproduction axis

The gut–reproduction axis is an emerging concept that links GM to reproductive function (Lin et al., 2025; Bao et al., 2026). Gut–reproduction axis refers to a bidirectional regulatory axis formed between the GM and the female reproductive system through pathways such as metabolites, immune responses, sex hormone metabolism, and intestinal barrier function. GM dysbiosis has been associated with systemic and local pelvic immune imbalance, enhanced inflammatory responses, and endocrine disorders, which may contribute to the pathogenesis and development of female reproductive system diseases, including PID. The homeostasis of the GM plays a crucial role in reproductive health, interacting with sex hormones at every stage of female reproductive health. On the one hand, the microbiota is correlated with female reproductive health and has a significant impact on it (Qi et al., 2021). GM dysbiosis has been associated with reduced levels of SCFAs and increased levels of LPS in animal models (Wang S. et al., 2022). GM and its metabolites affect female reproductive function by regulating endocrine homeostasis, immune balance, alleviating oxidative stress, and improving the inflammatory state (Nannini et al., 2025). Microbiome provides new insights for in-depth study of the etiology and mechanisms of PID, offers a broad application prospect for its diagnosis and treatment, and provides a new theoretical basis for the prevention and treatment of PID.

## 4 Microbial imbalance in PID

### 4.1 Imbalance of GM

**FIGURE 4:**
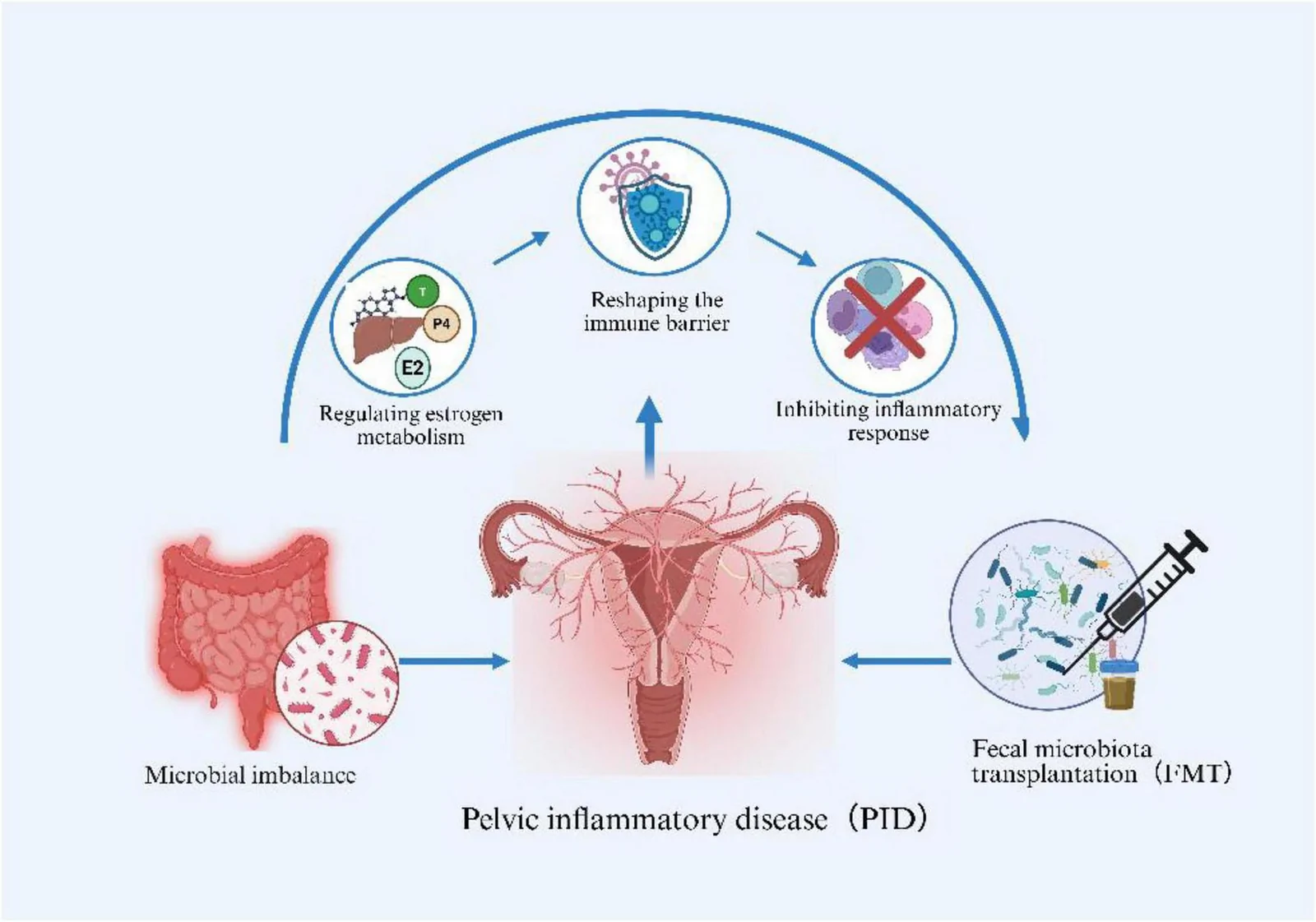
Regulating GM to alleviate PID. The GM influences the inflammatory susceptibility and tissue repair of the FRT by regulating estrogen metabolism, reshaping mucosal immune tolerance, and suppressing abnormal inflammatory responses. FMT can correct gut dysbiosis, restore the homeostatic regulatory role of the GM, and thereby alleviate PID. Created with BioRender.

The occurrence of PID is related to GM imbalance (Salliss et al., 2021; Dong et al., 2025; Figure 4). The stability of the intestinal barrier is the foundation of intestinal physiological function. Studies have found that changes in intestinal barrier function are associated with inflammatory diseases, often accompanied by increased intestinal permeability and microbiota translocation, triggering the production and release of inflammatory factors (Barone et al., 2023). Dysregulation of GM leads to damage to the intestinal villi, affecting the connections between intestinal cells, allowing LPS released by the microbiota to rapidly enter the body, activating the immune system and triggering inflammatory reactions (Ravel et al., 2021). PID patients with dysbacteriosis, leading to the translocation of bacteria and endotoxins in the intestine. Hu et al. found that the diversity and abundance of GM in mice with endometritis were reduced, the expression of SCFAs was low, and the phagocytosis and reactivity of neutrophils were weakened, making them unable to effectively clear pathogens, thus increasing the risk of inflammatory injury (Hu et al., 2020). Experimental findings indicate that the GM in PID rats undergoes significant disruption, characterized by altered relative abundances of major bacterial phyla, increased proportions of the genera *Lactobacillus* spp., *Clostridium* spp. and *Treponema* spp., and a marked reduction in overall bacterial diversity (Xue et al., 2023).

### 4.2 Imbalance of the reproductive microbiota

Research has shown that PID patients exhibit vaginal dysbiosis (Sweeney et al., 2022). Kim et al. detected multiple microorganisms in the vaginal secretions of PID patients, including *Ureaplasma urealyticum*, *Chlamydia trachomatis*, *Mycoplasma hominis*, *Mycoplasma genitalium*, *Trichomonas vaginalis*, and *Candida albicans*. The study also concluded that an imbalance in the vaginal microbiota is associated with an increased risk of PID, highlighting the importance of maintaining vaginal microbiota balance for PID prevention (Kim et al., 2021b). Research has found that mixed vaginitis causes more severe disruption to the vaginal microecological balance, with more complex and diverse manifestations. Diagnosis and treatment are significantly more difficult than single-pathogen vaginal infections. Mixed vaginitis is also an important reason for the failure of PID treatment and recurrent episodes (Turpin et al., 2021).

Related studies have reported that infections with *Ureaplasma urealyticum* and *Chlamydia trachomatis* are associated with indicators in vaginal secretions, such as peroxidase, sialidase, leukocyte esterase, and decreased *Lactobacillus* spp. (Miyoshi et al., 2022). The infection rates of young women with *Chlamydia trachomatis* and *Neisseria gonorrhoeae* have shown a significant upward trend. Filardo et al. found significant differences in the cervical microbiota between *Chlamydia trachomatis*-positive female patients and healthy women. The overall distribution of *Lactobacillus* spp. in *Chlamydia trachomatis*-positive patients decreased, while the overall distribution of anaerobic bacteria increased (Filardo et al., 2017). Endogenous pathogens such as *Escherichia coli* and *Bacteroides fragilis* reside in the vagina and are not normally pathogenic. An *in vitro* study by Nardini et al. demonstrated that *Lactobacillus crispatus* inhibits the infectivity of *Chlamydia trachomatis*, a major pathogen associated with PID. This finding suggests that *Lactobacillus crispatus* may contribute to the prevention of PID by reducing the infectivity of this pathogen (Nardini et al., 2016).

## 5 Mechanism of microbiota affecting PID

### 5.1 Microbial imbalance exacerbates hormonal disruption

Microecological imbalance affects sex hormone levels. Research has found that the density of *Lactobacillus* spp. in patients with vaginitis was positively correlated with the levels of testosterone and progesterone, both of which are lower than those in healthy subjects, suggesting that women with low testosterone and progesterone levels are prone to vaginal infections. Hormonal levels and menstrual cycle changes affect the vaginal microbiota and increase the risk of infection. A study has found that women aged 30-40 have the highest risk of recurrent PID, with more severe symptoms and poorer treatment outcomes (Kreisel et al., 2017). Abnormal levels of sex hormones lead to a weakened immune response in the reproductive tract and changes in the reproductive tract environment, increasing the risk of pathogen infection. Progesterone thickens cervical mucus, potentially enhancing the mechanical barrier against ascending pathogens, and may also modulate local immune function in the reproductive tract. Androgens are beneficial for the colonization of *Lactobacillus* spp. in vaginal epithelial cells, promoting the growth of *Lactobacillus* spp. and regulating and restoring the reproductive tract microenvironment (Berger et al., 2005). The estrogenome is the set of bacterial genomes in the GM that specifically metabolize estrogens (Ou et al., 2025). Estrogen regulates the balance and stability of the vaginal microbiota by maintaining a suitable acidic environment, sustaining the dominance of *Lactobacillus* spp., and modulating vaginal mucosal immunity.

Excessive or insufficient estrogen leads to changes in cell composition and their metabolites, affecting the immunity of vaginal mucosa, and altering the living environment of microorganisms. Research has found that GM regulates estrogen levels, with *Clostridia* spp. having the greatest impact on estrogen metabolism (Flores et al., 2012). The changes of *Clostridia* spp. are associated with estrogen metabolism, and estrogen inhibits the body’s oxidative stress response (Strehlow et al., 2003). Estrogen deficiency increases the susceptibility of the body to disease, further accelerating the release of inflammatory factors and exacerbating the inflammatory response in the local pelvic tissues.

### 5.2 Microbial imbalance exacerbates inflammation

Inflammatory response is the main pathological basis of PID. Continuous inflammatory stimulation disrupts the balance of the microbiome, forming a vicious cycle of infection, inflammation, and microbiome imbalance, which prolongs the course of PID. Gholiof et al. found that vaginal microbiota imbalance leads to increased levels of pro-inflammatory factors in the cervix and vaginal fluids, which not only raises the risk of infection but also affects overall reproductive health by accelerating inflammatory responses (Gholiof et al., 2022). The Yi-Qi-Qing-Shi-Hua-Yu method ameliorates uterine inflammation and fibrosis in rats with sequelae of PID, primarily by inhibiting the TLR4/NF-κB signaling pathway, suppressing inflammatory responses, regulating T cell proportions, and improving intestinal flora structure (Yin et al., 2025). Furthermore, both *in vivo* and *in vitro* studies using an LPS-induced endometritis model have demonstrated that miR-148a attenuates endometrial inflammation by blocking the activation of TLR4/NF-κB p65 and decreasing the levels of IL-1β and TNF-α (Jiang et al., 2020). GM-mediated activation of the aromatic hydrocarbon receptors repairs intestinal barrier homeostasis and inhibits inflammatory responses. *Lactobacillus* spp. are the main producers of aromatic hydrocarbon receptor ligands. Researchers have found that the abundance of *Lactobacillus* spp. in the GM of mice with endometritis is reduced, inhibiting the activation of aromatic hydrocarbon receptors and impairing intestinal barrier function, accelerating the rapid progression of inflammatory responses (Zhao et al., 2022). Dong et al. reported that in mice with acute endometritis, the relative abundance of opportunistic pathogens increased in the gut, while certain beneficial bacteria decreased. The study also found elevated expression of inflammatory cytokines in uterine tissue, such as IL-6, IL-1β, and TNF-α (Dong et al., 2021). LPS, a metabolite of GM, is a recognized pro-inflammatory factor. LPS from intestinal Gram-negative bacteria directly acts on endometrial and fallopian tube epithelial cells via the TLR4 pathway, induces endocytosis of tight junction proteins, increases paracellular permeability, and creates conditions for ascending infection of lower genital tract pathogens. When the microbiota is imbalanced, the levels of LPS increase, which bind to TLR4, induce the expression of TNF-α, and activate NF-κB, accelerating the production and secretion of inflammatory factors (Zhang et al., 2022). Gut-derived systemic LPS not only impairs epithelial barrier integrity but also triggers epithelial cell pyroptosis via the NLRP3 inflammasome, further destroying the structural integrity of the reproductive tract mucosa and forming an intestinal barrier disruption, reproductive tract barrier injury, and inflammation aggravation vicious cycle.

Research has found that the SCFAs in the feces of mice with endometritis are significantly reduced. SCFAs activate the anti-inflammatory signaling pathway in the body by binding to G protein-coupled receptors, and when their expression is low, they mediate the release of IL-6, leading to PID (Woods Acevedo and Pfeiffer, 2021). Tight junction proteins such as occludin and claudins are important indicators of intestinal barrier function and play a crucial role in preventing pathogen invasion (di Vito et al., 2022). Wang et al. found that *Escherichia coli* disrupts the barrier system by activating the TLR4/NF-κ B signaling pathway, reducing the expression of tight junction proteins ZO-1 and claudin-3, increasing intestinal wall permeability, and releasing inflammatory factors TNF-α and IL-1β into the bloodstream, inducing the occurrence of endometritis (Wang K. et al., 2022). Microbiota-derived butyrate from colonic fermentation can reach reproductive tract tissues through systemic circulation, directly upregulate tight junction protein expression in fallopian tube and endometrial epithelial cells via GPR109A, and reduce epithelial barrier permeability in *in vitro* human cell models. SCFAs also promote epithelial mucus secretion and enhance the mechanical barrier function of the reproductive tract PMID:40917749.

### 5.3 Microbial imbalance exacerbates immune imbalance

When PID patients have weakened immunity, pathogenic microorganisms inside the vagina and exogenous microorganisms trigger ascending infections, leading to recurrent disease. Immune dysfunction is the main pathogenesis of recurrent PID. The normal and balanced immune regulation function determines the immune homeostasis. Research has reported that in biopsy specimens from patients with PID caused by *Chlamydia* spp. Following infection, the phagocytic activity of neutrophils and macrophages is reduced, the production of SIgA is decreased, local immunity is disrupted, and the anti-infection function of natural killer cells is severely impaired. As the largest ‘immune organ’ in the body, the gut plays a crucial role in resisting pathogenic infections (Zhou et al., 2021). The GM as a key factor in maintaining intestinal homeostasis, which affects the immune function by regulating the homeostasis and development of immune cells (Muñoz et al., 2019). Metabolites derived from GM maintain the function of immune cells, enhance the body’s resistance to pathogenic microorganisms, form the GM-immune system axis (Rosser et al., 2020), stimulate the activity of B cells, secrete immunoglobulin IgA, bind with bacteria to form immune complexes, which are eventually phagocytosed and cleared by phagocytes, inhibiting the occurrence of inflammatory and autoimmune responses. Gut-derived SCFAs promote pelvic macrophage polarization to the M2 anti-inflammatory phenotype via the GPR43 receptor, increase IL-10 secretion, and reduce the release of TNF-α and IL-1β. In contrast, gut-derived LPS induces M1 polarization via TLR4, forming a pro-inflammatory positive feedback loop (Ashonibare et al., 2024).

## 6 Potential treatments for microbiome-based therapies in PID

In the treatment of PID, the antibiotic regimens currently recommended by guidelines are mainly based on the spectrum of pathogen coverage and drug sensitivity. Antibiotics kill bacteria and alleviate symptoms, but the pathological changes in local tissues and the sterile pelvic inflammation make antibiotics ineffective for treating PID. Long-term unreasonable use of antibiotics increases toxic side effects, raises the risk of resistance, and leads to recurrent disease (Breijyeh et al., 2020). Research has shown that long-term use of antibiotics leads to a microecological imbalance. There is an urgent need to seek new treatment (Tong et al., 2024). Ozone therapy has been reported to be relatively low in cost and associated with few adverse reactions in certain clinical applications. However, high-quality clinical evidence supporting its safety and efficacy specifically in the context of PID remains limited, and current understanding is largely derived from animal studies (Wei et al., 2018; Merhi et al., 2019).

### 6.1 Dietary fiber

Dietary fiber affects the reproductive microenvironment through the gut reproductive axis. Dietary fiber plays a role in the prevention of PID (Jin et al., 2025). Research has found that dietary fiber plays an important role in regulating systemic inflammation, immune function, and influencing GM (Makki et al., 2018; Shivakoti et al., 2022). A high-fiber diet promotes the growth of gut bacteria, whose metabolites exhibit potent anti-inflammatory and immunomodulatory properties (Chadchan et al., 2022). Women with higher dietary fiber intake have a lower risk of bacterial vaginosis (BV), as fiber plays a positive role in regulating vaginal microbiota and enhancing mucosal defense (Shivakoti et al., 2020).

The β-glucuronidase in the GM activates estrogen, and dietary fiber helps maintain microbial balance (Ervin et al., 2019). Activated estrogen increases the thickness of vaginal epithelium, promotes lactate production, enhances reproductive tract defense, and reduces the risk of infection (Baker et al., 2017). Consuming fiber-rich fruits and vegetables, as well as fermented foods, helps enhance microbial diversity and promotes the growth of beneficial bacteria such as *Lactobacillus* spp. and *Bifidobacterium* spp. (Feng and Liu, 2022). Omega-3 PUFAs, as essential fatty acids in the body, participate in the regulation of intestinal homeostasis (Costantini et al., 2017). Higher intake of copper and magnesium helps reduce the incidence of PID (Hu et al., 2023; Chen et al., 2024). High-fat diets promote inflammatory diseases by increasing pro-inflammatory factors and macrophage accumulation (Herup-Wheeler et al., 2024).

### 6.2 Microecologics

Microecologies are crucial for maintaining microbial balance (Mei and Li, 2022). Probiotics effectively inhibit the growth of pathogenic bacteria and maintain the balance of GM (Hill et al., 2014). Supplementing with specific probiotics, such as *Lactobacillus* spp. and *Bifidobacterium* spp., enhance intestinal health, improve immune function, and benefit reproductive health (Jang et al., 2017; Kadogami et al., 2020). The lactate produced by *Lactobacillus* spp. helps maintain normal vaginal epithelial cell integrity and inhibits the growth and reproduction of pathogenic vaginal bacteria (Huang et al., 2023). Patients with BV who take *Lactobacillus rhamnosus* significantly improve vaginal microbiota and increase the recovery rate from BV (Reznichenko et al., 2020). Prebiotics are a type of organic substance that is not digested or absorbed by the host, selectively promotes the metabolism and proliferation of beneficial bacteria in the body, and improves the health of the host (Blaut, 2002). Clinical trials have reported that prebiotic vaginal gels improve the therapeutic effect of BV (Hakimi et al., 2018). As a composite preparation of probiotics and prebiotics, Synbiotics also show similar therapeutic effects (Swanson et al., 2020). The above studies indicate that microecologies have potential applications in reproductive health, providing new research directions for the treatment of PID.

### 6.3 Microbiota transplantation

Microbiota transplantation refers to the transfer of microbiota from a healthy donor to a recipient, which is a novel therapeutic method for restoring microbial balance (Łaniewski et al., 2020). Fecal microbiota transplantation may offer a potential direction for future research on female reproductive system diseases. It is important to note that most existing evidence on microbiota-based therapies for reproductive tract disorders comes from studies on BV or endometritis, rather than directly from patients with PID. Nevertheless, this approach remains a potential future strategy for restoring reproductive function in PID. Hu et al. conducted fecal microbiota transplantation to evaluate the relationship between GM and endometritis, and found that GM had a protective effect on endometritis induced by *Staphylococcus aureus* by regulating the level of SCFAs and maintaining the phagocytic ability and responsiveness of neutrophils (Hu et al., 2020). Endometritis and PID both involve inflammation of the reproductive system, providing experimental support for the potential role of GM in PID. Lev-Sagie et al. found that vaginal microbiome transplantation alleviated BV-related symptoms and restored *Lactobacillus* spp. dominance in the vagina. However, it should be noted that this approach remains experimental, and clinical evidence is still limited (Lev-Sagie et al., 2019). Kutteh et al. found that intestinal immunity induces the secretion of specific antibodies in FRT secretions, and this protective humoral and cellular immune response is of great significance for the prevention of sexually transmitted diseases (Kutteh et al., 2001). Gut commensal *norank_f_Muribaculaceae* produces guanosine, which enters uterine tissue through the circulation, directly upregulates CXCL14 expression in uterine epithelial cells, and enhances epithelial anti-infection ability. Fecal microbiota transplantation experiments confirmed that this GM-metabolite-epithelial axis exerts a significant protective effect against *E. coli*-induced endometritis. The protective effect is completely abolished after GM depletion by antibiotics, providing direct causal evidence for the gut–reproduction axis PMID:40227949.

### 6.4 Herbs

Herbs are characterized by multiple molecules, multiple targets, and a low likelihood of developing drug resistance, which has a unique effect on regulating and improving the microbiota of PID patients. Herbs stimulate blood circulation, enhance cell membrane permeability, and accelerate the absorption of inflammation. Ji et al. found that Hongteng Baijiang Decoction reduced the abundance of *Clostridium* spp., and *Ruminococcus* spp. in the intestines of a rat model of PID, restored GM balance and alleviated pelvic tissue inflammation and damage in these animals (Ji et al., 2024). These findings provide experimental support for the potential role of microbiota modulation in alleviating PID-related inflammation. Herbs have been reported to modulate GM composition and diversity and restore intestinal barrier function. Based on these findings, herbs may also alleviate pelvic tissue inflammation and PID damage through similar mechanisms. BAO et al. found that mild moxibustion significantly increased the relative DNA abundance of probiotics in the GM of rats with irritable bowel syndrome while reducing *Escherichia coli* (Bao et al., 2019). Although irritable bowel syndrome and PID are different clinical entities, they share common pathophysiological features, including low-grade chronic inflammation and immune response changes. Therefore, evidence of effective microbiota modulation and intestinal barrier repair in irritable bowel syndrome models may provide relevant insights into the pathophysiology of PID. Ma et al. demonstrated that total glucosides of paeony attenuates chronic endometritis in rats by restoring gut microbial homeostasis and inhibiting the TLR4/NF-κB/NLRP3 inflammatory cascade, providing preclinical evidence for the gut–reproduction axis in pelvic inflammatory pathology PMID:42432693. Zhang et al. reported that rosemary extract mitigates oviduct dysfunction in laying hens via enhancing intestinal barrier integrity, reshaping cecal microbiota, promoting butyrate production, and preserving immune homeostasis, highlighting a GM-mediated regulatory axis for reproductive tract function (Zhang et al., 2023).

## 7 Conclusion

The high incidence of PID and its potential long-term impact on female reproductive health remain important clinical concerns. Dysbiosis has been associated with pelvic organ dysfunction through pathways such as altered hormone levels, inflammatory factor regulation, and immune homeostasis. These mechanisms may interact with each other and potentially influence female reproductive health via the gut–reproduction axis, but direct causal evidence in PID is still limited. Current evidence is largely associative or derived from experimental models. With advances in microbiome research, combining microbiota-based therapies with conventional antibiotics has emerged as a potential approach to enhance treatment efficacy, strengthen barrier function, and reduce side effects. The microbiome holds promise for providing new insights into the diagnosis and treatment of PID. However, there are still many challenges to overcome: ➀Which microbial communities indicate that the FRT is in a state of “microbial imbalance”? Microbiome profiles vary substantially across PID subtypes, and inconsistent patient stratification across studies has led to discrepant conclusions. ➁The pelvic anatomy is complex, and microbial metabolites affect the synthesis and metabolism of substances through multiple signaling pathways. The specific mechanisms of their role in the occurrence and development of PID need to be further explored. ➂The causal relationship between human gut dysbiosis and PID remains unclear; most clinical evidence is cross-sectional. Although animal studies provide support for a causal link, the bidirectional interactions between the gut and reproductive tract microbiomes in PID remain largely uncharacterized. ➃How the highly individualized microbiome guides universal PID treatment. In the future, we will further strengthen multidisciplinary collaboration, integrating expertise from microbiology, obstetrics and gynecology, immunology, and pharmacy, to comprehensively improve diagnosis and treatment, ultimately promoting the improvement of female reproductive health.

## References

[B1] Aljadah M. Widlansky M. E. (2023). Finding needles in the gut microbiota’s haystack. *Circ. Res*. 132 182–184. 10.1161/CIRCRESAHA.122.322354 36656969 PMC9869460

[B2] Ashonibare V. J. Akorede B. A. Ashonibare P. J. Akhigbe T. M. Akhigbe R. E. (2024). Gut microbiota-gonadal axis: the impact of gut microbiota on reproductive functions. *Front. Immunol*. 15:1346035. 10.3389/fimmu.2024.1346035 38482009 PMC10933031

[B3] Baker J. M. Al-Nakkash L. Herbst-Kralovetz M. M. (2017). Estrogen-gut microbiome axis: physiological and clinical implications. *Maturitas* 103 45–53. 10.1016/j.maturitas.2017.06.025 28778332

[B4] Bao C. H. Wang C. Y. Li G. N. Yan Y. L. Wang D. Jin X. M. et al.. (2019). Effect of mild moxibustion on intestinal microbiota and NLRP6 inflammasome signaling in rats with post-inflammatory irritable bowel syndrome. *World J. Gastroenterol*. 25 4696–4714. 10.3748/wjg.v25.i32.4696 31528095 PMC6718040

[B5] Bao Y. Hu X. Gao T. Hua W. (2026). Association between dietary index for gut microbiota and the risk of pelvic inflammatory disease: mediating role of anti- and pro-inflammatory dietary patterns. *Food Sci. Nutr*. 14:e71563. 10.1002/fsn3.71563 41728037 PMC12920683

[B6] Barone M. Ramayo-Caldas Y. Estellé J. Tambosco K. Chadi S. Maillard F. et al.. (2023). Gut barrier-microbiota imbalances in early life lead to higher sensitivity to inflammation in a murine model of C-section delivery. *Microbiome* 11:140. 10.1186/s40168-023-01584-0 37394428 PMC10316582

[B7] Barrientos-Durán A. Fuentes-López A. de Salazar A. Plaza-Díaz J. García F. (2020). Reviewing the composition of vaginal microbiota: inclusion of nutrition and probiotic factors in the maintenance of eubiosis. *Nutrients* 12:419. 10.3390/nu12020419 32041107 PMC7071153

[B8] Bartlett E. C. Levison W. B. Munday P. E. (2013). Pelvic inflammatory disease. *BMJ* 346:f3189. 10.1136/bmj.f3189 23704128

[B9] Berger L. El-Alfy M. Martel C. Labrie F. (2005). Effects of dehydroepiandrosterone, premarin and Acolbifene on histomorphology and sex steroid receptors in the rat vagina. *J. Steroid Biochem. Mol. Biol*. 96 201–215. 10.1016/j.jsbmb.2005.02.018 15979306

[B10] Blaut M. (2002). Relationship of prebiotics and food to intestinal microflora. *Eur. J. Nutr*. 41 (Suppl. 1), I11–I16. 10.1007/s00394-002-1102-7 12420111

[B11] Breijyeh Z. Jubeh B. Karaman R. (2020). Resistance of gram-negative bacteria to current antibacterial agents and approaches to resolve it. *Molecules* 25:1340. 10.3390/molecules25061340 32187986 PMC7144564

[B12] Brunham R. C. Gottlieb S. L. Paavonen J. (2015). Pelvic inflammatory disease. *N. Engl. J. Med*. 372 2039–2048. 10.1056/NEJMra1411426 25992748

[B13] Causer L. Liu B. Watts C. McManus H. Donovan B. Ward J. et al.. (2022). Hospitalisations for pelvic inflammatory disease in young Aboriginal women living in remote Australia: the role of chlamydia and gonorrhoea. *Sex Transm. Infect*. 98 445–447. 10.1136/sextrans-2021-055242 34887352

[B14] Chadchan S. B. Popli P. Ambati C. R. Tycksen E. Han S. J. Bulun S. E. et al.. (2021). Gut microbiota-derived short-chain fatty acids protect against the progression of endometriosis. *Life Sci. Alliance*. 4:e202101224. 10.26508/lsa.202101224 34593556 PMC8500332

[B15] Chadchan S. B. Singh V. Kommagani R. (2022). Female reproductive dysfunctions and the gut microbiota. *J. Mol. Endocrinol*. 69 R81–R94. 10.1530/JME-21-0238 35900833 PMC10031513

[B16] Chen C. Song X. Wei W. Zhong H. Dai J. Lan Z. et al.. (2017). The microbiota continuum along the female reproductive tract and its relation to uterine-related diseases. *Nat. Commun*. 8:875. 10.1038/s41467-017-00901-0 29042534 PMC5645390

[B17] Chen Y. Zhou J. Wang L. (2021). Role and mechanism of gut microbiota in human disease. *Front. Cell Infect. Microbiol*. 11:625913. 10.3389/fcimb.2021.625913 33816335 PMC8010197

[B18] Chen Z. Wu Z. Zhang Y. (2024). Association between dietary magnesium intake and pelvic inflammatory disease in US women: a cross-sectional study of NHANES. *Front. Nutr*. 11:1430730. 10.3389/fnut.2024.1430730 39171114 PMC11335488

[B19] Costantini L. Molinari R. Farinon B. Merendino N. (2017). Impact of omega-3 fatty acids on the gut microbiota. *Int. J. Mol. Sci*. 18:2645. 10.3390/ijms18122645 29215589 PMC5751248

[B20] Darville T. (2021). Pelvic inflammatory disease due to *Neisseria gonorrhoeae* and *Chlamydia trachomatis*: immune evasion mechanisms and pathogenic disease pathways. *J. Infect. Dis*. 224 S39–S46. 10.1093/infdis/jiab031 34396413 PMC8365118

[B21] Day M. J. Spiteri G. Jacobsson S. Woodford N. Amato-Gauci A. J. Cole M. J. et al.. (2018). Stably high azithromycin resistance and decreasing ceftriaxone susceptibility in *Neisseria gonorrhoeae* in 25 European countries, 2016. *BMC Infect. Dis*. 18:609. 10.1186/s12879-018-3528-4 30509194 PMC6276195

[B22] de Vos W. M. Tilg H. Van Hul M. Cani P. D. (2022). Gut microbiome and health: mechanistic insights. *Gut* 71 1020–1032. 10.1136/gutjnl-2021-326789 35105664 PMC8995832

[B23] Deka N. Hassan S. Seghal Kiran G. Selvin J. (2021). Insights into the role of vaginal microbiome in women’s health. *J. Basic Microbiol*. 61 1071–1084. 10.1002/jobm.202100421 34763361

[B24] Den Heijer C. D. J. Hoebe C. J. P. A. Driessen J. H. M. Wolffs P. Van Den Broek I. V. F. Hoenderboom B. M. et al.. (2020). Erratum to: *Chlamydia trachomatis* and the risk of pelvic inflammatory disease, ectopic pregnancy, and female infertility: a retrospective cohort study among primary care patients. *Clin. Infect. Dis*. 70:2459. 10.1093/cid/ciz897 31535123 PMC7245142

[B25] di Vito R. Conte C. Traina G. (2022). A multi-strain probiotic formulation improves intestinal barrier function by the modulation of tight and adherent junction proteins. *Cells* 11:2617. 10.3390/cells11162617 36010692 PMC9406415

[B26] Dong S. Du Y. Wang H. Yuan W. Ai W. Liu L. (2025). Research progress on the interaction between intestinal flora and microRNA in pelvic inflammatory diseases. *Noncoding RNA Res*. 11 303–312. 10.1016/j.ncrna.2025.01.007 39931541 PMC11808595

[B27] Dong Y. Yuan Y. Ma Y. Luo Y. Zhou W. Deng X. et al.. (2021). Combined intestinal metabolomics and microbiota analysis for acute endometritis induced by lipopolysaccharide in mice. *Front. Cell Infect. Microbiol*. 11:791373. 10.3389/fcimb.2021.791373 34976866 PMC8718680

[B28] Ervin S. M. Li H. Lim L. Roberts L. R. Liang X. Mani S. et al.. (2019). Gut microbial β-glucuronidases reactivate estrogens as components of the estrobolome that reactivate estrogens. *J. Biol. Chem*. 294 18586–18599. 10.1074/jbc.RA119.010950 31636122 PMC6901331

[B29] Fang R. L. Chen L. X. Shu W. S. Yao S. Z. Wang S. W. Chen Y. Q. (2016). Barcoded sequencing reveals diverse intrauterine microbiomes in patients suffering with endometrial polyps. *Am. J. Transl. Res.* 8 1581–1592. 27186283 PMC4859642

[B30] Feng T. Liu Y. (2022). Microorganisms in the reproductive system and probiotic’s regulatory effects on reproductive health. *Comput. Struct. Biotechnol. J*. 20 1541–1553. 10.1016/j.csbj.2022.03.017 35465162 PMC9010680

[B31] Filardo S. Di Pietro M. Porpora M. G. Recine N. Farcomeni A. Latino M. A. et al.. (2017). Diversity of cervical microbiota in asymptomatic *Chlamydia trachomatis* genital infection: a pilot study. *Front. Cell Infect. Microbiol*. 7:321. 10.3389/fcimb.2017.00321 28770172 PMC5509768

[B32] Flores R. Shi J. Fuhrman B. Xu X. Veenstra T. D. Gail M. H. et al.. (2012). Fecal microbial determinants of fecal and systemic estrogens and estrogen metabolites: a cross-sectional study. *J. Transl. Med*. 10:253. 10.1186/1479-5876-10-253 23259758 PMC3552825

[B33] Gajer P. Brotman R. M. Bai G. Sakamoto J. Schütte U. M. Zhong X. et al.. (2012). Temporal dynamics of the human vaginal microbiota. *Sci. Transl. Med.* 4:132ra52. 10.1126/scitranslmed.3003605 22553250 PMC3722878

[B34] Gao Y. Wang X. Wang Q. Jiang L. Wu C. Guo Y. et al.. (2025). Global impact of pelvic inflammatory diseases attributable to sexually transmitted infections in women of childbearing age: a comprehensive analysis based on GBD 2021 data and bibliometric analysis. *Int. J. Surg.* [Online ahead of print]. 10.1097/JS9.0000000000004066 41247816

[B35] Garrett W. S. (2015). Cancer and the microbiota. *Science* 348 80–86. 10.1126/science.aaa4972 25838377 PMC5535753

[B36] Geva-Zatorsky N. Sefik E. Kua L. Pasman L. Tan T. G. Ortiz-Lopez A. et al.. (2017). Mining the human gut microbiota for immunomodulatory organisms. *Cell* 168 928–943.e11. 10.1016/j.cell.2017.01.022 28215708 PMC7774263

[B37] Gholiof M. Adamson-De Luca E. Wessels J. M. (2022). The female reproductive tract microbiotas, inflammation, and gynecological conditions. *Front. Reprod. Health* 4:963752. 10.3389/frph.2022.963752 36303679 PMC9580710

[B38] Greydanus D. E. Cabral M. D. Patel D. R. (2022). Pelvic inflammatory disease in the adolescent and young adult: an update. *Dis. Mon*. 68:101287. 10.1016/j.disamonth.2021.101287 34521505

[B39] Greydanus D. E. Dodich C. (2015). Pelvic inflammatory disease in the adolescent: a poignant, perplexing, potentially preventable problem for patients and physicians. *Curr. Opin. Pediatr*. 27 92–99. 10.1097/MOP.0000000000000183 25514575

[B40] Greydanus D. E. Merrick J. Cabral M. D. (2018). Pelvic inflammatory disease: management requires a patient, prudent, prejudice-free provider. *Int. J. Adolesc. Med. Health* 32:/j/ijamh.2020.32.issue-1/ijamh-2018-0216/ijamh-2018-0216.xml. 10.1515/ijamh-2018-0216 30422800

[B41] Ha M. M. Belcher H. M. E. Butz A. M. Perin J. Matson P. A. Trent M. (2019). Partner notification, treatment, and subsequent condom use after pelvic inflammatory disease: implications for dyadic intervention with urban youth. *Clin. Pediatr*. 58 1271–1276. 10.1177/0009922819852979 31165630 PMC6868422

[B42] Hakimi S. Farhan F. Farshbaf-Khalili A. Dehghan P. Javadzadeh Y. Abbasalizadeh S. et al.. (2018). The effect of prebiotic vaginal gel with adjuvant oral metronidazole tablets on treatment and recurrence of bacterial vaginosis: a triple-blind randomized controlled study. *Arch. Gynecol. Obstet*. 297 109–116. 10.1007/s00404-017-4555-x 28983665

[B43] Herup-Wheeler T. Shi M. Harvey M. E. Talwar C. Kommagani R. MacLean J. A. et al.. (2024). High-fat diets promote peritoneal inflammation and augment endometriosis-associated abdominal hyperalgesia. *Front. Endocrinol*. 15:1336496. 10.3389/fendo.2024.1336496 38559689 PMC10978581

[B44] Hill C. Guarner F. Reid G. Gibson G. R. Merenstein D. J. Pot B. et al.. (2014). Expert consensus document. The International Scientific Association for Probiotics and Prebiotics consensus statement on the scope and appropriate use of the term probiotic. *Nat. Rev. Gastroenterol. Hepatol*. 11 506–514. 10.1038/nrgastro.2014.66 24912386

[B45] Hu P. Zhang S. Li H. Yan X. Zhang X. Zhang Q. (2023). Association between dietary trace minerals and pelvic inflammatory disease: data from the 2015-2018 National Health and Nutrition Examination Surveys. *Front. Nutr*. 10:1273509. 10.3389/fnut.2023.1273509 38089925 PMC10715429

[B46] Hu X. Mu R. Xu M. Yuan X. Jiang P. Guo J. et al.. (2020). Gut microbiota mediate the protective effects on endometritis induced by *Staphylococcus aureus* in mice. *Food Funct*. 11 3695–3705. 10.1039/c9fo02963j 32307472

[B47] Huang T. Cao R. Liu P. Liu J. Yu X. (2022). The severity of depression is associated with pelvic inflammatory diseases: a cross-sectional study of the United States National Health and Nutrition Examinations from 2013 to 2018. *Front. Med.* 9:926351. 10.3389/fmed.2022.926351 36314030 PMC9596754

[B48] Huang Y. P. Shi J. Y. Luo S. C. Xu S. Y. Zhang J. D. Molnár I. et al.. (2023). Antimicrobial substances and mechanisms of *Lactobacillus rhamnosus* against *Gardnerella vaginalis*. *Probiot. Antimicrob Proteins* 15 400–410. 10.1007/s12602-022-10019-5 36459386

[B49] Jang S. E. Jeong J. J. Choi S. Y. Kim H. Han M. J. Kim D. H. (2017). *Lactobacillus rhamnosus* HN001 and *Lactobacillus acidophilus* La-14 attenuate Gardnerella vaginalis-infected bacterial vaginosis in mice. *Nutrients* 9:531. 10.3390/nu9060531 28545241 PMC5490510

[B50] Jenkins S. M. Vadakekut E. S. (2025). *Pelvic Inflammatory Disease.* Treasure Island, FL: StatPearls. 29763134

[B51] Ji X. Hu Q. Yang C. Huang L. Huang Y. Deng L. et al.. (2024). Modified Hongteng Baijiang decoction enema improves sequelae of pelvic inflammatory disease by regulating the LIF/JAK2/STAT3 pathway and gut microbiota. *Immun. Inflamm. Dis*. 12:e1300. 10.1002/iid3.1300 38896093 PMC11186298

[B52] Jiang K. Yang J. Song C. He F. Yang L. Li X. (2021). Enforced expression of miR-92b blunts *E. coli* lipopolysaccharide-mediated inflammatory injury by activating the PI3K/AKT/β-catenin pathway via targeting PTEN. *Int. J. Biol. Sci*. 17 1289–1301. 10.7150/ijbs.56933 33867846 PMC8040465

[B53] Jiang K. Yang J. Yang C. Zhang T. Shaukat A. Yang X. et al.. (2020). miR-148a suppresses inflammation in lipopolysaccharide-induced endometritis. *J. Cell Mol. Med*. 24 405–417. 10.1111/jcmm.14744 31756048 PMC6933404

[B54] Jin H. Niu Z. Zhao X. (2025). The association between dietary fiber intake and pelvic inflammatory disease in women: findings from the NHANES 2015-2018. *BMC Womens Health* 25:356. 10.1186/s12905-025-03911-z 40682044 PMC12273326

[B55] Kadogami D. Nakaoka Y. Morimoto Y. (2020). Use of a vaginal probiotic suppository and antibiotics to influence the composition of the endometrial microbiota. *Reprod. Biol*. 20 307–314. 10.1016/j.repbio.2020.07.001 32680750

[B56] Kim S. Seo H. Rahim M. A. Lee S. Kim Y. S. Song H. Y. (2021a). Changes in the microbiome of vaginal fluid after menopause in Korean women. *J. Microbiol. Biotechnol*. 31 1490–1500. 10.4014/jmb.2106.06022 34489372 PMC9705842

[B57] Kim S. Seo H. Rahim M. A. Tajdozian H. Kim Y. S. Song H. Y. (2021b). Characteristics of vaginal microbiome in women with pelvic inflammatory disease in Korea. *Pol. J. Microbiol*. 70 345–357. 10.33073/pjm-2021-033 34584529 PMC8458998

[B58] Koedooder R. Mackens S. Budding A. Fares D. Blockeel C. Laven J. et al.. (2019). Identification and evaluation of the microbiome in the female and male reproductive tracts. *Hum. Reprod. Update* 25 298–325. 10.1093/humupd/dmy048 30938752

[B59] Kreisel K. Torrone E. Bernstein K. Hong J. Gorwitz R. (2017). Prevalence of pelvic inflammatory disease in sexually experienced women of reproductive Age - United States, 2013-2014. *MMWR Morb Mortal Wkly. Rep*. 66 80–83. 10.15585/mmwr.mm6603a3 28125569 PMC5573882

[B60] Kreisel K. M. Llata E. Haderxhanaj L. Pearson W. S. Tao G. Wiesenfeld H. C. et al.. (2021). The burden of and trends in pelvic inflammatory disease in the United States, 2006-2016. *J. Infect. Dis*. 224 S103–S112. 10.1093/infdis/jiaa771 34396411 PMC10243492

[B61] Kutteh W. H. Kantele A. Moldoveanu Z. Crowley-Nowick P. A. Mestecky J. (2001). Induction of specific immune responses in the genital tract of women after oral or rectal immunization and rectal boosting with *Salmonella typhi* Ty 21a vaccine. *J. Reprod. Immunol*. 52 61–75. 10.1016/s0165-0378(01)00109-7 11600178

[B62] Łaniewski P. Ilhan Z. E. Herbst-Kralovetz M. M. (2020). The microbiome and gynaecological cancer development, prevention and therapy. *Nat. Rev. Urol*. 17 232–250. 10.1038/s41585-020-0286-z 32071434 PMC9977514

[B63] Lee S. A. Tsai H. T. Ou H. C. Han C. P. Tee Y. T. Chen Y. C. et al.. (2008). Plasma interleukin-1beta, -6, -8 and tumor necrosis factor-alpha as highly informative markers of pelvic inflammatory disease. *Clin. Chem. Lab. Med*. 46 997–1003. 10.1515/CCLM.2008.196 18624621

[B64] Lev-Sagie A. Goldman-Wohl D. Cohen Y. Dori-Bachash M. Leshem A. Mor U. et al.. (2019). Vaginal microbiome transplantation in women with intractable bacterial vaginosis. *Nat. Med*. 25 1500–1504. 10.1038/s41591-019-0600-6 31591599

[B65] Liang J. Li M. Zhang L. Yang Y. Jin X. Zhang Q. et al.. (2023). Analysis of the microbiota composition in the genital tract of infertile patients with chronic endometritis or endometrial polyps. *Front. Cell Infect. Microbiol*. 13:1125640. 10.3389/fcimb.2023.1125640 37284497 PMC10240353

[B66] Lin X. Yu Z. Liu Y. Li C. Hu H. Hu J. C. et al.. (2025). Gut-X axis. *Imeta* 4:e270. 10.1002/imt2.270 40027477 PMC11865426

[B67] Lloyd-Price J. Abu-Ali G. Huttenhower C. (2016). The healthy human microbiome. *Genome Med*. 8:51. 10.1186/s13073-016-0307-y 27122046 PMC4848870

[B68] Loeper N. Graspeuntner S. Rupp J. (2018). Microbiota changes impact on sexually transmitted infections and the development of pelvic inflammatory disease. *Microbes Infect*. 20 505–511. 10.1016/j.micinf.2018.02.003 29452257

[B69] Lykke M. R. Becher N. Haahr T. Boedtkjer E. Jensen J. S. Uldbjerg N. (2021). Vaginal, cervical and uterine pH in women with normal and abnormal vaginal microbiota. *Pathogens* 10:90. 10.3390/pathogens10020090 33498288 PMC7909242

[B70] Mackeen A. D. Packard R. E. Ota E. Speer L. (2015). Antibiotic regimens for postpartum endometritis. *Cochrane Database Syst. Rev*. 2015:CD001067. 10.1002/14651858.CD001067.pub3 25922861 PMC7050613

[B71] Makki K. Deehan E. C. Walter J. Bäckhed F. (2018). The impact of dietary fiber on gut microbiota in host health and disease. *Cell Host Microbe* 23 705–715. 10.1016/j.chom.2018.05.012 29902436

[B72] Martino C. Dilmore A. H. Burcham Z. M. Metcalf J. L. Jeste D. Knight R. (2022). Microbiota succession throughout life from the cradle to the grave. *Nat. Rev. Microbiol*. 20 707–720. 10.1038/s41579-022-00768-z 35906422 PMC12875531

[B73] Mei Z. Li D. (2022). The role of probiotics in vaginal health. *Front. Cell Infect. Microbiol*. 12:963868. 10.3389/fcimb.2022.963868 35967876 PMC9366906

[B74] Merhi Z. Garg B. Moseley-LaRue R. Moseley A. R. Smith A. H. Zhang J. (2019). Ozone therapy: a potential therapeutic adjunct for improving female reproductive health. *Med. Gas Res*. 9 101–105. 10.4103/2045-9912.260652 31249259 PMC6607862

[B75] Mitchell C. Prabhu M. (2013). Pelvic inflammatory disease: current concepts in pathogenesis, diagnosis and treatment. *Infect. Dis. Clin. North Am*. 27 793–809. 10.1016/j.idc.2013.08.004 24275271 PMC3843151

[B76] Miyoshi Y. Suga S. Sugimi S. Kurata N. Yamashita H. Yasuhi I. (2022). Vaginal Ureaplasma urealyticum or *Mycoplasma hominis* and preterm delivery in women with threatened preterm labor. *J. Matern Fetal Neonatal Med*. 35 878–883. 10.1080/14767058.2020.1733517 32131651

[B77] Muñoz L. Borrero M. J. Úbeda M. Conde E. Del Campo R. Rodríguez-Serrano M. et al.. (2019). Intestinal immune dysregulation driven by dysbiosis promotes barrier disruption and bacterial translocation in rats with cirrhosis. *Hepatology* 70 925–938. 10.1002/hep.30349 30414342

[B78] Murray S. Augustyniak M. Murase J. E. Fischer-Betz R. Nelson-Piercy C. Peniuta M. et al.. (2021). Barriers to shared decision-making with women of reproductive age affected by a chronic inflammatory disease: a mixed-methods needs assessment of dermatologists and rheumatologists. *BMJ Open* 11:e043960. 10.1136/bmjopen-2020-043960 34135086 PMC8212186

[B79] Nakahira E. S. Maximiano L. F. Lima F. R. Ussami E. Y. (2017). Abdominal and pelvic actinomycosis due to longstanding intrauterine device: a slow and devastating infection. *Autops Case Rep*. 7 43–47. 10.4322/acr.2017.001 28536687 PMC5436921

[B80] Nannini G. Cei F. Amedei A. (2025). Unraveling the contribution of estrobolome alterations to endometriosis pathogenesis. *Curr. Issues Mol. Biol*. 47:502. 10.3390/cimb47070502 40728971 PMC12293932

[B81] Nardini P. Ñahui Palomino R. A. Parolin C. Laghi L. Foschi C. Cevenini R. et al.. (2016). *Lactobacillus crispatus* inhibits the infectivity of *Chlamydia trachomatis* elementary bodies, in vitro study. *Sci. Rep*. 6:29024. 10.1038/srep29024 27354249 PMC4926251

[B82] Nkwabong E. Dingom M. A. (2015). Acute pelvic inflammatory disease in cameroon: a cross sectional descriptive study. *Afr. J. Reprod. Health* 19 87–91. 27337857

[B83] Noyes N. Cho K. C. Ravel J. Forney L. J. Abdo Z. (2018). Associations between sexual habits, menstrual hygiene practices, demographics and the vaginal microbiome as revealed by Bayesian network analysis. *PLoS One* 13:e0191625. 10.1371/journal.pone.0191625 29364944 PMC5783405

[B84] Ou Y. Wang H. Zhou C. Chen Y. Lyu J. Feng M. et al.. (2025). Endometriosis-associated infertility: multi-omics insights into pathogenesis and precision therapeutics. *Front. Endocrinol*. 16:1613334. 10.3389/fendo.2025.1613334 41113721 PMC12527851

[B85] Porcari S. Mullish B. H. Asnicar F. Ng S. C. Zhao L. Hansen R. et al.. (2025). International consensus statement on microbiome testing in clinical practice. *Lancet Gastroenterol. Hepatol*. 10 154–167. 10.1016/S2468-1253(24)00311-X 39647502 PMC12343204

[B86] Qi X. Yun C. Pang Y. Qiao J. (2021). The impact of the gut microbiota on the reproductive and metabolic endocrine system. *Gut Microbes* 13 1–21. 10.1080/19490976.2021.1894070 33722164 PMC7971312

[B87] Qin J. Li R. Raes J. Arumugam M. Burgdorf K. S. Manichanh C. et al.. (2010). A human gut microbial gene catalogue established by metagenomic sequencing. *Nature* 464 59–65. 10.1038/nature08821 20203603 PMC3779803

[B88] Ravel J. Gajer P. Abdo Z. Schneider G. M. Koenig S. S. McCulle S. L. et al.. (2011). Vaginal microbiome of reproductive-age women. *Proc. Natl. Acad. Sci. U. S. A*. 108 (Suppl. 1), 4680–4687. 10.1073/pnas.1002611107 20534435 PMC3063603

[B89] Ravel J. Moreno I. Simón C. (2021). Bacterial vaginosis and its association with infertility, endometritis, and pelvic inflammatory disease. *Am. J. Obstet Gynecol*. 224 251–257. 10.1016/j.ajog.2020.10.019 33091407

[B90] Reznichenko H. Henyk N. Maliuk V. Khyzhnyak T. Tynna Y. Filipiuk I. et al.. (2020). Oral intake of lactobacilli can be helpful in symptomatic bacterial vaginosis: a randomized clinical study. *J. Low Genit. Tract. Dis*. 24 284–289. 10.1097/LGT.0000000000000518 32091443

[B91] Ross J. Guaschino S. Cusini M. Jensen J. (2018). 2017 European guideline for the management of pelvic inflammatory disease. *Int. J. Std. AIDS* 29 108–114. 10.1177/0956462417744099 29198181

[B92] Ross J. Judlin P. Jensen J. (2014). 2012 European guideline for the management of pelvic inflammatory disease. *Int. J. Std. AIDS* 25 1–7. 10.1177/0956462413498714 24216035

[B93] Rosser E. C. Piper C. J. M. Matei D. E. Blair P. A. Rendeiro A. F. Orford M. et al.. (2020). Microbiota-derived metabolites suppress arthritis by amplifying aryl-hydrocarbon receptor activation in regulatory B Cells. *Cell Metab.* 31 837–851.e10. 10.1016/j.cmet.2020.03.003 32213346 PMC7156916

[B94] Saad M. J. Santos A. Prada P. O. (2016). Linking gut microbiota and inflammation to obesity and insulin resistance. *Physiology* 31 283–293. 10.1152/physiol.00041.2015 27252163

[B95] Safrai M. Rottenstreich A. Shushan A. Gilad R. Benshushan A. Levin G. (2020). Risk factors for recurrent Pelvic inflammatory disease. *Eur. J. Obstet Gynecol. Reprod. Biol*. 244 40–44. 10.1016/j.ejogrb.2019.11.004 31734623

[B96] Salliss M. E. Farland L. V. Mahnert N. D. Herbst-Kralovetz M. M. (2021). The role of gut and genital microbiota and the estrobolome in endometriosis, infertility and chronic pelvic pain. *Hum. Reprod. Update* 28 92–131. 10.1093/humupd/dmab035 34718567

[B97] Savaris R. F. Fuhrich D. G. Duarte R. V. Franik S. Ross J. D. C. (2019). Antibiotic therapy for pelvic inflammatory disease: an abridged version of a Cochrane systematic review and meta-analysis of randomised controlled trials. *Sex Transm. Infect*. 95 21–27. 10.1136/sextrans-2018-053693 30341232 PMC6580736

[B98] Savaris R. F. Fuhrich D. G. Maissiat J. Duarte R. V. Ross J. (2020). Antibiotic therapy for pelvic inflammatory disease. *Cochrane Database Syst. Rev*. 8:CD010285. 10.1002/14651858.CD010285.pub3 32820536 PMC8094882

[B99] Sen P. Molinero-Perez A. O’Riordan K. J. McCafferty C. P. O’Halloran K. D. Cryan J. F. (2021). Microbiota and sleep: awakening the gut feeling. *Trends Mol. Med*. 27 935–945. 10.1016/j.molmed.2021.07.004 34364787

[B100] Shen C. C. Yang A. C. Hung J. H. Hu L. Y. Chiang Y. Y. Tsai S. J. (2016). Risk of psychiatric disorders following pelvic inflammatory disease: a nationwide population-based retrospective cohort study. *J. Psychosom. Obstet. Gynaecol*. 37 6–11. 10.3109/0167482X.2015.1124852 26821967

[B101] Shivakoti R. Biggs M. L. Djoussé L. Durda P. J. Kizer J. R. Psaty B. et al.. (2022). Intake and sources of dietary fiber, inflammation, and cardiovascular disease in older US Adults. *JAMA Netw. Open* 5:e225012. 10.1001/jamanetworkopen.2022.5012 35357453 PMC8972036

[B102] Shivakoti R. Tuddenham S. Caulfield L. E. Murphy C. Robinson C. Ravel J. et al.. (2020). Dietary macronutrient intake and molecular-bacterial vaginosis: role of fiber. *Clin. Nutr*. 39 3066–3071. 10.1016/j.clnu.2020.01.011 32033845 PMC7387193

[B103] Siegenthaler F. Krause E. Mueller M. D. (2020). [Management of pelvic inflammatory disease]. *Ther Umsch* 77 164–170. 10.1024/0040-5930/a001171 32772692

[B104] Sonnex C. (2010). Toll-like receptors and genital tract infection. *Int. J. STD AIDS* 21 153–157. 10.1258/ijsa.2009.009525 20215617

[B105] Soper D. E. (2010). Pelvic inflammatory disease. *Obstet. Gynecol*. 116 419–428. 10.1097/AOG.0b013e3181e92c54 20664404

[B106] Strehlow K. Rotter S. Wassmann S. Adam O. Grohé C. Laufs K. et al.. (2003). Modulation of antioxidant enzyme expression and function by estrogen. *Circ. Res*. 93 170–177. 10.1161/01.RES.0000082334.17947.11 12816884

[B107] Swanson K. S. Gibson G. R. Hutkins R. Reimer R. A. Reid G. Verbeke K. et al.. (2020). The International Scientific Association for Probiotics and Prebiotics (ISAPP) consensus statement on the definition and scope of synbiotics. *Nat. Rev. Gastroenterol. Hepatol*. 17 687–701. 10.1038/s41575-020-0344-2 32826966 PMC7581511

[B108] Sweeney S. Bateson D. Fleming K. Huston W. (2022). Factors associated with pelvic inflammatory disease: a case series analysis of family planning clinic data. *Womens Health* 18:17455057221112263. 10.1177/17455057221112263 35819075 PMC9280787

[B109] Taira T. Broussard N. Bugg C. (2022). Pelvic inflammatory disease: diagnosis and treatment in the emergency department. *Emerg. Med. Pract.* 24 1–24. 36378827

[B110] Tamarelle J. Thiébaut A. C. M. de Barbeyrac B. Bébéar C. Ravel J. Delarocque-Astagneau E. (2019). The vaginal microbiota and its association with human papillomavirus, *Chlamydia trachomatis*, *Neisseria gonorrhoeae* and *Mycoplasma genitalium* infections: a systematic review and meta-analysis. *Clin. Microbiol. Infect*. 25 35–47. 10.1016/j.cmi.2018.04.019 29729331 PMC7362580

[B111] Taylor B. D. Darville T. Ferrell R. E. Ness R. B. Haggerty C. L. (2013). Racial variation in toll-like receptor variants among women with pelvic inflammatory disease. *J. Infect. Dis*. 207 940–946. 10.1093/infdis/jis922 23255565 PMC3571443

[B112] Taylor B. D. Ness R. B. Darville T. Haggerty C. L. (2011). Microbial correlates of delayed care for pelvic inflammatory disease. *Sex. Transm. Dis*. 38 434–438. 10.1097/OLQ.0b013e3181ffa7c7 21124257 PMC3657731

[B113] Tepper N. K. Steenland M. W. Gaffield M. E. Marchbanks P. A. Curtis K. M. (2013). Retention of intrauterine devices in women who acquire pelvic inflammatory disease: a systematic review. *Contraception* 87 655–660. 10.1016/j.contraception.2012.08.011 23040135

[B114] Tong G. Qian H. Li D. Li J. Chen J. Li X. et al.. (2024). Intestinal flora imbalance induced by antibiotic use in rats. *J. Inflamm. Res*. 17 1789–1804. 10.2147/JIR.S447098 38528993 PMC10961240

[B115] Torcia M. G. (2019). Interplay among vaginal microbiome, immune response and sexually transmitted viral infections. *Int. J. Mol. Sci*. 20:266. 10.3390/ijms20020266 30641869 PMC6359169

[B116] Turpin R. Tuddenham S. He X. Klebanoff M. A. Ghanem K. G. Brotman R. M. (2021). Bacterial vaginosis and behavioral factors associated with incident pelvic inflammatory disease in the longitudinal study of vaginal flora. *J. Infect. Dis*. 224 S137–S144. 10.1093/infdis/jiab103 34396403 PMC8499701

[B117] van de Wijgert J. H. Borgdorff H. Verhelst R. Crucitti T. Francis S. Verstraelen H. et al.. (2014). The vaginal microbiota: what have we learned after a decade of molecular characterization? *PLoS One* 9:e105998. 10.1371/journal.pone.0105998 25148517 PMC4141851

[B118] Wang K. Wang K. Wang J. Yu F. Ye C. (2022). Protective effect of *Clostridium butyricum* on *Escherichia coli*-induced endometritis in mice via ameliorating endometrial barrier and inhibiting inflammatory response. *Microbiol. Spectr*. 10:e0328622. 10.1128/spectrum.03286-22 36321897 PMC9769554

[B119] Wang S. Fu W. Zhao X. Chang X. Liu H. Zhou L. et al.. (2022). Zearalenone disturbs the reproductive-immune axis in pigs: the role of gut microbial metabolites. *Microbiome* 10:234. 10.1186/s40168-022-01397-7 36536466 PMC9762105

[B120] Wee B. A. Thomas M. Sweeney E. L. Frentiu F. D. Samios M. Ravel J. et al.. (2018). A retrospective pilot study to determine whether the reproductive tract microbiota differs between women with a history of infertility and fertile women. *Aust. N. Z. J. Obstet Gynaecol*. 58 341–348. 10.1111/ajo.12754 29280134

[B121] Wei A. Feng H. Jia X. M. Tang H. Liao Y. Y. Li B. R. (2018). Ozone therapy ameliorates inflammation and endometrial injury in rats with pelvic inflammatory disease. *Biomed. Pharmacother*. 107 1418–1425. 10.1016/j.biopha.2018.07.137 30257358

[B122] White B. A. Creedon D. J. Nelson K. E. Wilson B. A. (2011). The vaginal microbiome in health and disease. *Trends Endocrinol. Metab*. 22 389–393. 10.1016/j.tem.2011.06.001 21757370 PMC3183339

[B123] Wiesenfeld H. C. Meyn L. A. Darville T. Macio I. S. Hillier S. L. (2021). A randomized controlled trial of ceftriaxone and doxycycline, with or without metronidazole, for the treatment of acute pelvic inflammatory disease. *Clin. Infect. Dis*. 72 1181–1189. 10.1093/cid/ciaa101 32052831 PMC8028096

[B124] Woods Acevedo M. A. Pfeiffer J. K. (2021). Microbiota-immune system interactions and enteric virus infection. *Curr. Opin. Virol*. 46 15–19. 10.1016/j.coviro.2020.08.005 32898729 PMC7933313

[B125] Xholli A. Cannoletta M. Cagnacci A. (2014). Seasonal trend of acute pelvic inflammatory disease. *Arch. Gynecol. Obstet*. 289 1017–1022. 10.1007/s00404-013-3094-3 24258789

[B126] Xu C. Yi M. Xiao Z. Xiang F. Wu M. Zhang Z. et al.. (2025). New idea of Fuke Qianjin capsule in treating sequelae of pelvic inflammatory disease: anti-inflammatory in the early stage and reparative in the later stage. *J. Ethnopharmacol*. 338:119066. 10.1016/j.jep.2024.119066 39528116

[B127] Xue G. Zheng Z. Liang X. Zheng Y. Wu H. (2023). Uterine tissue metabonomics combined with 16S rRNA gene sequencing to analyze the changes of gut microbiota in mice with endometritis and the intervention effect of tau interferon. *Microbiol. Spectr*. 11:e0040923. 10.1128/spectrum.00409-23 37067455 PMC10269590

[B128] Yagur Y. Weitzner O. Barchilon Tiosano L. Paitan Y. Katzir M. Schonman R. et al.. (2021). Characteristics of pelvic inflammatory disease caused by sexually transmitted disease - An epidemiologic study. *J. Gynecol. Obstet Hum. Reprod*. 50:102176. 10.1016/j.jogoh.2021.102176 34087450

[B129] Yang X. Zhang X. Yang W. Yu H. He Q. Xu H. et al.. (2021). Gut microbiota in adipose tissue dysfunction induced cardiovascular disease: role as a metabolic organ. *Front. Endocrinol*. 12:749125. 10.3389/fendo.2021.749125 34552566 PMC8450894

[B130] Yates J. A. Collis O. A. Sueblinvong T. Collis T. K. (2017). Red snappers and red herrings: pelvic tuberculosis causing elevated CA 125 and mimicking advanced ovarian cancer. a case report and literature review. *Hawaii J. Med. Public Health* 76 220–224. 28808611 PMC5551276

[B131] Yin X. Wang Y. Li M. Zhang E. Huang L. Yang C. (2025). Yi-Qi-Qing-Shi-Hua-Yu method improves uterine inflammation in rats with sequelae of pelvic inflammatory disease through the TLR4/NF-κB signaling pathway and regulates intestinal flora. *Tissue Cell* 95:102918. 10.1016/j.tice.2025.102918 40253799

[B132] You J. S. Kim M. J. Chung H. S. Chung Y. E. Park I. Chung S. P. et al.. (2012). Clinical features of Fitz-Hugh-Curtis Syndrome in the emergency department. *Yonsei Med. J*. 53 753–758. 10.3349/ymj.2012.53.4.753 22665342 PMC3381477

[B133] Yudin M. H. Hillier S. L. Wiesenfeld H. C. Krohn M. A. Amortegui A. A. Sweet R. L. (2003). Vaginal polymorphonuclear leukocytes and bacterial vaginosis as markers for histologic endometritis among women without symptoms of pelvic inflammatory disease. *Am. J. Obstet. Gynecol*. 188 318–323. 10.1067/mob.2003.105 12592233

[B134] Zhang L. Ge J. Gao F. Yang M. Li H. Xia F. et al.. (2023). Rosemary extract improves egg quality by altering gut barrier function, intestinal microbiota and oviductal gene expressions in late-phase laying hens. *J. Anim. Sci. Biotechnol*. 14:121. 10.1186/s40104-023-00904-6 37667318 PMC10476401

[B135] Zhang X. Chen B. D. Zhao L. D. Li H. (2020). The gut microbiota: emerging evidence in autoimmune diseases. *Trends Mol. Med*. 26 862–873. 10.1016/j.molmed.2020.04.001 32402849

[B136] Zhang Y. Duan X. Wassie T. Wang H. H. Li T. Xie C. et al.. (2022). Enteromorpha prolifera polysaccharide-zinc complex modulates the immune response and alleviates LPS-induced intestinal inflammation via inhibiting the TLR4/NF-κB signaling pathway. *Food Funct*. 13 52–63. 10.1039/d1fo02171k 34704575

[B137] Zhao C. Bao L. Qiu M. Feng L. Chen L. Liu Z. et al.. (2022). Dietary tryptophan-mediated aryl hydrocarbon receptor activation by the gut microbiota alleviates *Escherichia coli*-induced endometritis in mice. *Microbiol. Spectr*. 10:e0081122. 10.1128/spectrum.00811-22 35727038 PMC9430277

[B138] Zhao Q. Chen Y. Huang W. Zhou H. Zhang W. (2023). Drug-microbiota interactions: an emerging priority for precision medicine. *Signal Transduct. Target Ther*. 8:386. 10.1038/s41392-023-01619-w 37806986 PMC10560686

[B139] Zhou C. B. Zhou Y. L. Fang J. Y. (2021). Gut microbiota in cancer immune response and immunotherapy. *Trends Cancer* 7 647–660. 10.1016/j.trecan.2021.01.010 33674230

